# 1,3-Bis(2-cyano­benz­yl)imidazolium bromide

**DOI:** 10.1107/S1600536811048951

**Published:** 2011-11-30

**Authors:** Rosenani A. Haque, Safaa A. Ahmed, Zulikha H. Zetty, Madhukar Hemamalini, Hoong-Kun Fun

**Affiliations:** a School of Chemical Sciences, Universiti Sains Malaysia, 11800 USM, Penang, Malaysia; b Department of Chemistry, College of Education Samarra, University of Tikrit, Tikrit 43001, Iraq; c X-ray Crystallography Unit, School of Physics, Universiti Sains Malaysia, 11800 USM, Penang, Malaysia

## Abstract

In the title salt, C_19_H_15_N_4_^+^·Br^−^, the central imidazole ring makes dihedral angles of 83.1 (2) and 87.6 (2)° with the terminal benzene rings. The dihedral angle between the terminal benzene rings is 6.77 (19)°; the cyanide substituents have an *anti* orientation. In the crystal, the cations and anions are linked *via* C—H⋯N and C—H⋯Br hydrogen bonds, forming sheets lying parallel to the *ac* plane.

## Related literature

For details and applications of *N*-heterocylic carbene, see: Wanzlick & Kleiner (1961 ▶); Fahlbusch *et al.* (2009 ▶); Demir *et al.* (2009 ▶); Grasa *et al.* (2002 ▶); Buchowicz *et al.* (2006 ▶); Marko *et al.* (2002 ▶).
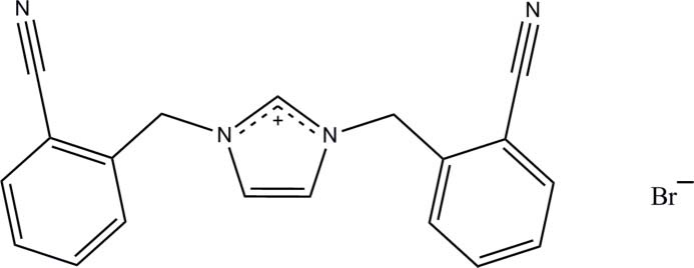


## Experimental

### 

#### Crystal data

C_19_H_15_N_4_^+^·Br^−^
*M* *_r_* = 379.26
Monoclinic, 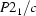

*a* = 9.0661 (9) Å
*b* = 8.0357 (9) Å
*c* = 24.697 (3) Å
β = 95.651 (2)°
*V* = 1790.5 (3) Å^3^
*Z* = 4
Mo *K*α radiation
μ = 2.30 mm^−1^
*T* = 296 K
0.36 × 0.17 × 0.10 mm

#### Data collection

Bruker APEXII DUO CCD diffractometer
Absorption correction: multi-scan (*SADABS*; Bruker, 2009 ▶) *T*_min_ = 0.494, *T*_max_ = 0.799
12922 measured reflections
4066 independent reflections
2486 reflections with *I* > 2σ(*I*)
*R*_int_ = 0.034

#### Refinement

*R*[*F* ^2^ > 2σ(*F* ^2^)] = 0.049
*wR*(*F* ^2^) = 0.149
*S* = 1.02
4066 reflections
217 parameters
H-atom parameters constrained
Δρ_max_ = 1.14 e Å^−3^
Δρ_min_ = −0.82 e Å^−3^

### 

Data collection: *APEX2* (Bruker, 2009 ▶); cell refinement: *SAINT* (Bruker, 2009 ▶); data reduction: *SAINT*; program(s) used to solve structure: *SHELXTL* (Sheldrick, 2008 ▶); program(s) used to refine structure: *SHELXTL*; molecular graphics: *SHELXTL*; software used to prepare material for publication: *SHELXTL* and *PLATON* (Spek, 2009 ▶).

## Supplementary Material

Crystal structure: contains datablock(s) global, I. DOI: 10.1107/S1600536811048951/hb6514sup1.cif

Structure factors: contains datablock(s) I. DOI: 10.1107/S1600536811048951/hb6514Isup2.hkl

Supplementary material file. DOI: 10.1107/S1600536811048951/hb6514Isup3.cml

Additional supplementary materials:  crystallographic information; 3D view; checkCIF report

## Figures and Tables

**Table 1:** Hydrogen-bond geometry (Å, °)

*D*—H⋯*A*	*D*—H	H⋯*A*	*D*⋯*A*	*D*—H⋯*A*
C1—H1*A*⋯Br1^i^	0.93	2.70	3.531 (4)	149
C2—H2*A*⋯Br1^ii^	0.93	2.67	3.579 (4)	165
C3—H3*A*⋯N4^iii^	0.93	2.50	3.377 (6)	157
C4—H4*B*⋯Br1^i^	0.97	2.86	3.730 (4)	149
C7—H7*A*⋯N4^iv^	0.93	2.60	3.390 (5)	144
C10—H10*A*⋯Br1^v^	0.93	2.88	3.678 (4)	144

Symmetry codes: (i) 
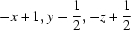
; (ii) 
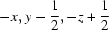
; (iii) 
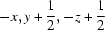
; (iv) 
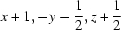
; (v) 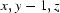.

**Fig. 1.**
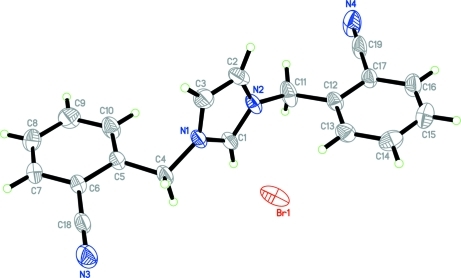
The asymmetric unit of the title compound, showing 30% probability displacement.

**Fig. 2.**
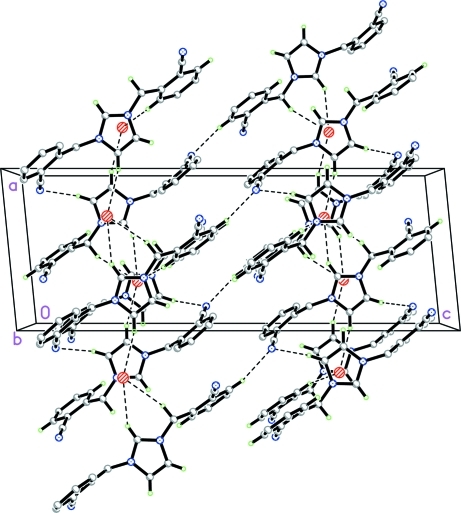
The crystal packing of the title compound, showing hydrogen-bonded (dashed lines) network. H atoms not involved in hydrogen bond interactions are omitted for clarity.

## supplementary crystallographic information

### Comment

Since the investigation of *N*-heterocyclic carbene (NHC) chemistry by Wanzlick and Kleiner (1961), NHCs have played a major role as ligands in coordination and organometallic chemistry (Fahlbusch *et al.*, 2009). During the past decades it has been proven as an alternative to tertiary phosphines in homogeneous catalysis. Due to NHC's strong σ-donating and negligible π-accepting characters, they are compatible with metals in a variety of oxidation states. NHC can stabilize catalytically active intermediates (Demir *et al.*, 2009) making it a very versatile ligand system. NHC complexes with every transition metal are now known and their applications especially in the area of catalysis cover a broad spectrum such as hydroboration (Grasa *et al.*, 2002), polymerization reactions (Buchowicz *et al.*, 2006) and hydrosilation (Marko *et al.*, 2002). Furthermore, NHCs are easy to handle, stable and inexpensive resulting in their receiving a great deal of interest compared to other types of carbenes.

In (I), the asymmetric unit contains a 1,3-Bis(2-cyanobenzyl)imidazolium cation and a bromide anion.  The central imidazole (N1,N2/C1–C3) ring makes dihedral angles of  83.1 (2) and 87.6 (2)° with the terminal phenyl (C5–C10 and C12–C17) rings. The dihedral angle between the two terminal phenyl (C5–C10 and C12–C17) rings is 6.77 (19)°.

In the crystal, (Fig. 2), the cations and anions are linked *via* C—H···N and C—H···Br hydrogen bonds (Table 1), forming two-dimensional networks parallel to the *ac*-plane.

### Experimental

Imidazole (0.3 g, 3.7 mmol) and potassium hydroxide (0.2 g, 5.5 mmol) was stirred for 2 h in 25 mL of ethanol. 2-Bromomethyl benzonitrile (1.8 g, 9.2 mmol) was then added and the mixture was refluxed at 80°C for 24 h. The resulting clear crystals were isolated by decantation, washed with fresh n-hexane (2 X 3 ml) and then left to dry at ambient temperature. Yield: 1.3 g, (94%); m.p: 233–234°C. Colourless blocks were obtained by slow evaporation of the salt solution in ethanol at ambient temperature.

### Refinement

All hydrogen atoms were positioned geometrically [ C–H = 0.93 or 0.97 Å] and were refined using a riding model, with *U*_iso_(H) = 1.2 *U*_eq_(C).

### Crystal data

**Table:**

C_19_H_15_N_4_^+^·Br^−^	*F*(000) = 768
*M**_r_* = 379.26	*D*_x_ = 1.407 Mg m^−^^3^
Monoclinic, *P*2_1_/*c*	Mo *K*α radiation, λ = 0.71073 Å
Hall symbol: -P 2ybc	Cell parameters from 2803 reflections
*a* = 9.0661 (9) Å	θ = 2.9–23.8°
*b* = 8.0357 (9) Å	µ = 2.30 mm^−^^1^
*c* = 24.697 (3) Å	*T* = 296 K
β = 95.651 (2)°	Block, colourless
*V* = 1790.5 (3) Å^3^	0.36 × 0.17 × 0.10 mm
*Z* = 4	

### Data collection

**Table:**

Bruker APEXII DUO CCD diffractometer	4066 independent reflections
Radiation source: fine-focus sealed tube	2486 reflections with *I* > 2σ(*I*)
graphite	*R*_int_ = 0.034
φ and ω scans	θ_max_ = 27.5°, θ_min_ = 1.7°
Absorption correction: multi-scan (*SADABS*; Bruker, 2009)	*h* = −11→11
*T*_min_ = 0.494, *T*_max_ = 0.799	*k* = −10→10
12922 measured reflections	*l* = −32→32

### Refinement

**Table:**

Refinement on *F*^2^	Primary atom site location: structure-invariant direct methods
Least-squares matrix: full	Secondary atom site location: difference Fourier map
*R*[*F*^2^ > 2σ(*F*^2^)] = 0.049	Hydrogen site location: inferred from neighbouring sites
*wR*(*F*^2^) = 0.149	H-atom parameters constrained
*S* = 1.02	*w* = 1/[σ^2^(*F*_o_^2^) + (0.0749*P*)^2^ + 0.6395*P*] where *P* = (*F*_o_^2^ + 2*F*_c_^2^)/3
4066 reflections	(Δ/σ)_max_ = 0.001
217 parameters	Δρ_max_ = 1.14 e Å^−^^3^
0 restraints	Δρ_min_ = −0.82 e Å^−^^3^

### Special details

**Table:**

Geometry. All s.u.'s (except the s.u. in the dihedral angle between two l.s. planes) are estimated using the full covariance matrix. The cell s.u.'s are taken into account individually in the estimation of s.u.'s in distances, angles and torsion angles; correlations between s.u.'s in cell parameters are only used when they are defined by crystal symmetry. An approximate (isotropic) treatment of cell s.u.'s is used for estimating s.u.'s involving l.s. planes.
Refinement. Refinement of F^2^ against ALL reflections. The weighted R-factor wR and goodness of fit S are based on F^2^, conventional R-factors R are based on F, with F set to zero for negative F^2^. The threshold expression of F^2^ > 2σ(F^2^) is used only for calculating R-factors(gt) etc. and is not relevant to the choice of reflections for refinement. R-factors based on F^2^ are statistically about twice as large as those based on F, and R- factors based on ALL data will be even larger.

### Fractional atomic coordinates and isotropic or equivalent isotropic displacement parameters (Å^2^)

**Table:**

	*x*	*y*	*z*	*U*_iso_*/*U*_eq_	
Br1	0.29310 (4)	0.46183 (7)	0.271846 (19)	0.0744 (2)	
N1	0.3270 (3)	0.0222 (4)	0.29878 (11)	0.0487 (7)	
N2	0.1865 (3)	−0.0421 (4)	0.22727 (11)	0.0521 (8)	
N3	0.6850 (4)	0.2721 (5)	0.43842 (14)	0.0763 (11)	
N4	−0.1238 (5)	−0.2711 (6)	0.07253 (15)	0.0977 (15)	
C1	0.3264 (4)	−0.0178 (5)	0.24721 (13)	0.0482 (8)	
H1A	0.4092	−0.0274	0.2280	0.058*	
C2	0.0970 (4)	−0.0140 (6)	0.26727 (16)	0.0659 (11)	
H2A	−0.0058	−0.0215	0.2643	0.079*	
C3	0.1858 (4)	0.0268 (6)	0.31198 (15)	0.0628 (11)	
H3A	0.1557	0.0534	0.3459	0.075*	
C4	0.4592 (4)	0.0639 (5)	0.33548 (14)	0.0570 (10)	
H4A	0.4438	0.1698	0.3529	0.068*	
H4B	0.5435	0.0756	0.3144	0.068*	
C5	0.4932 (4)	−0.0676 (5)	0.37865 (13)	0.0467 (8)	
C6	0.5839 (4)	−0.0257 (5)	0.42592 (13)	0.0466 (8)	
C7	0.6238 (4)	−0.1459 (6)	0.46540 (14)	0.0605 (10)	
H7A	0.6816	−0.1167	0.4972	0.073*	
C8	0.5779 (5)	−0.3068 (6)	0.45731 (17)	0.0709 (12)	
H8A	0.6076	−0.3878	0.4830	0.085*	
C9	0.4881 (5)	−0.3487 (6)	0.41142 (19)	0.0741 (12)	
H9A	0.4544	−0.4575	0.4067	0.089*	
C10	0.4474 (5)	−0.2307 (6)	0.37226 (16)	0.0657 (11)	
H10A	0.3881	−0.2616	0.3410	0.079*	
C11	0.1370 (5)	−0.0897 (6)	0.17153 (16)	0.0749 (13)	
H11A	0.0594	−0.1727	0.1720	0.090*	
H11B	0.2191	−0.1401	0.1552	0.090*	
C12	0.0786 (4)	0.0566 (5)	0.13661 (13)	0.0507 (9)	
C13	0.1252 (4)	0.2175 (5)	0.14572 (15)	0.0579 (10)	
H13A	0.1924	0.2412	0.1756	0.070*	
C14	0.0744 (5)	0.3443 (6)	0.11149 (17)	0.0676 (11)	
H14A	0.1077	0.4524	0.1183	0.081*	
C15	−0.0260 (5)	0.3115 (6)	0.06708 (17)	0.0740 (12)	
H15A	−0.0618	0.3978	0.0444	0.089*	
C16	−0.0729 (5)	0.1509 (6)	0.05649 (16)	0.0693 (12)	
H16A	−0.1383	0.1278	0.0260	0.083*	
C17	−0.0230 (4)	0.0254 (5)	0.09104 (13)	0.0524 (9)	
C18	0.6389 (4)	0.1393 (6)	0.43334 (13)	0.0540 (9)	
C19	−0.0785 (5)	−0.1402 (6)	0.08064 (14)	0.0674 (12)	

### Atomic displacement parameters (Å^2^)

**Table:**

	*U*^11^	*U*^22^	*U*^33^	*U*^12^	*U*^13^	*U*^23^
Br1	0.0396 (2)	0.0842 (4)	0.0975 (4)	−0.00728 (19)	−0.00305 (18)	−0.0309 (2)
N1	0.0396 (14)	0.062 (2)	0.0417 (14)	−0.0013 (13)	−0.0096 (11)	−0.0008 (13)
N2	0.0481 (16)	0.054 (2)	0.0491 (15)	0.0021 (14)	−0.0196 (13)	−0.0085 (13)
N3	0.092 (3)	0.064 (3)	0.068 (2)	−0.018 (2)	−0.0216 (18)	−0.0005 (19)
N4	0.145 (4)	0.074 (3)	0.063 (2)	−0.033 (3)	−0.044 (2)	0.004 (2)
C1	0.0362 (15)	0.062 (3)	0.0448 (17)	0.0039 (15)	−0.0063 (13)	−0.0014 (16)
C2	0.0366 (17)	0.087 (3)	0.073 (2)	−0.0036 (18)	−0.0027 (16)	0.001 (2)
C3	0.054 (2)	0.083 (3)	0.0516 (19)	−0.003 (2)	0.0099 (16)	−0.001 (2)
C4	0.058 (2)	0.056 (3)	0.0509 (19)	−0.0101 (18)	−0.0213 (16)	−0.0008 (17)
C5	0.0467 (17)	0.045 (2)	0.0457 (17)	0.0022 (15)	−0.0080 (13)	−0.0016 (15)
C6	0.0464 (17)	0.052 (2)	0.0402 (16)	0.0005 (16)	−0.0039 (13)	−0.0024 (15)
C7	0.063 (2)	0.071 (3)	0.0456 (19)	0.000 (2)	−0.0081 (15)	0.0106 (18)
C8	0.075 (3)	0.065 (3)	0.071 (3)	0.008 (2)	−0.003 (2)	0.025 (2)
C9	0.083 (3)	0.042 (3)	0.095 (3)	−0.009 (2)	−0.004 (2)	0.005 (2)
C10	0.074 (3)	0.055 (3)	0.063 (2)	−0.002 (2)	−0.0172 (19)	−0.007 (2)
C11	0.095 (3)	0.058 (3)	0.062 (2)	0.010 (2)	−0.037 (2)	−0.014 (2)
C12	0.0511 (18)	0.052 (3)	0.0458 (17)	0.0003 (17)	−0.0088 (14)	−0.0069 (16)
C13	0.053 (2)	0.055 (3)	0.064 (2)	−0.0027 (18)	−0.0027 (16)	−0.009 (2)
C14	0.078 (3)	0.049 (3)	0.078 (3)	−0.007 (2)	0.022 (2)	−0.007 (2)
C15	0.095 (3)	0.063 (3)	0.065 (2)	0.017 (3)	0.008 (2)	0.014 (2)
C16	0.082 (3)	0.069 (3)	0.053 (2)	0.000 (2)	−0.0129 (19)	0.009 (2)
C17	0.059 (2)	0.054 (2)	0.0423 (17)	0.0001 (18)	−0.0079 (14)	0.0008 (16)
C18	0.058 (2)	0.059 (3)	0.0417 (17)	−0.0030 (19)	−0.0140 (15)	0.0000 (17)
C19	0.090 (3)	0.060 (3)	0.045 (2)	−0.006 (2)	−0.0303 (19)	0.0019 (19)

### Geometric parameters (Å, °)

**Table:**

N1—C1	1.313 (4)	C7—H7A	0.9300
N1—C3	1.352 (5)	C8—C9	1.371 (6)
N1—C4	1.469 (4)	C8—H8A	0.9300
N2—C1	1.329 (4)	C9—C10	1.378 (6)
N2—C2	1.358 (5)	C9—H9A	0.9300
N2—C11	1.456 (4)	C10—H10A	0.9300
N3—C18	1.148 (5)	C11—C12	1.522 (6)
N4—C19	1.140 (6)	C11—H11A	0.9700
C1—H1A	0.9300	C11—H11B	0.9700
C2—C3	1.341 (5)	C12—C13	1.372 (5)
C2—H2A	0.9300	C12—C17	1.405 (4)
C3—H3A	0.9300	C13—C14	1.374 (6)
C4—C5	1.511 (5)	C13—H13A	0.9300
C4—H4A	0.9700	C14—C15	1.380 (6)
C4—H4B	0.9700	C14—H14A	0.9300
C5—C10	1.380 (6)	C15—C16	1.376 (7)
C5—C6	1.401 (4)	C15—H15A	0.9300
C6—C7	1.395 (5)	C16—C17	1.369 (5)
C6—C18	1.422 (6)	C16—H16A	0.9300
C7—C8	1.366 (6)	C17—C19	1.437 (6)
			
C1—N1—C3	109.1 (3)	C8—C9—C10	120.5 (4)
C1—N1—C4	125.4 (3)	C8—C9—H9A	119.8
C3—N1—C4	125.4 (3)	C10—C9—H9A	119.8
C1—N2—C2	108.8 (3)	C9—C10—C5	121.1 (3)
C1—N2—C11	125.7 (3)	C9—C10—H10A	119.5
C2—N2—C11	125.5 (3)	C5—C10—H10A	119.5
N1—C1—N2	107.9 (3)	N2—C11—C12	113.0 (3)
N1—C1—H1A	126.0	N2—C11—H11A	109.0
N2—C1—H1A	126.0	C12—C11—H11A	109.0
C3—C2—N2	106.7 (3)	N2—C11—H11B	109.0
C3—C2—H2A	126.7	C12—C11—H11B	109.0
N2—C2—H2A	126.7	H11A—C11—H11B	107.8
C2—C3—N1	107.5 (3)	C13—C12—C17	117.8 (3)
C2—C3—H3A	126.3	C13—C12—C11	123.4 (3)
N1—C3—H3A	126.3	C17—C12—C11	118.7 (3)
N1—C4—C5	111.9 (3)	C12—C13—C14	121.2 (3)
N1—C4—H4A	109.2	C12—C13—H13A	119.4
C5—C4—H4A	109.2	C14—C13—H13A	119.4
N1—C4—H4B	109.2	C13—C14—C15	120.2 (4)
C5—C4—H4B	109.2	C13—C14—H14A	119.9
H4A—C4—H4B	107.9	C15—C14—H14A	119.9
C10—C5—C6	118.0 (3)	C16—C15—C14	119.8 (4)
C10—C5—C4	123.0 (3)	C16—C15—H15A	120.1
C6—C5—C4	118.8 (3)	C14—C15—H15A	120.1
C7—C6—C5	120.5 (4)	C17—C16—C15	119.7 (4)
C7—C6—C18	119.4 (3)	C17—C16—H16A	120.1
C5—C6—C18	120.1 (3)	C15—C16—H16A	120.1
C8—C7—C6	119.9 (3)	C16—C17—C12	121.2 (4)
C8—C7—H7A	120.1	C16—C17—C19	118.9 (3)
C6—C7—H7A	120.1	C12—C17—C19	119.9 (3)
C7—C8—C9	120.1 (4)	N3—C18—C6	178.6 (4)
C7—C8—H8A	120.0	N4—C19—C17	179.3 (6)
C9—C8—H8A	120.0		
			
C3—N1—C1—N2	−1.2 (4)	C7—C8—C9—C10	2.2 (7)
C4—N1—C1—N2	−178.2 (3)	C8—C9—C10—C5	−1.3 (7)
C2—N2—C1—N1	1.0 (4)	C6—C5—C10—C9	0.7 (6)
C11—N2—C1—N1	−179.5 (4)	C4—C5—C10—C9	176.2 (4)
C1—N2—C2—C3	−0.4 (5)	C1—N2—C11—C12	−100.4 (5)
C11—N2—C2—C3	−179.9 (4)	C2—N2—C11—C12	79.0 (5)
N2—C2—C3—N1	−0.3 (5)	N2—C11—C12—C13	28.0 (6)
C1—N1—C3—C2	0.9 (5)	N2—C11—C12—C17	−155.2 (4)
C4—N1—C3—C2	177.9 (4)	C17—C12—C13—C14	0.0 (6)
C1—N1—C4—C5	−111.6 (4)	C11—C12—C13—C14	176.8 (4)
C3—N1—C4—C5	71.9 (5)	C12—C13—C14—C15	0.3 (6)
N1—C4—C5—C10	24.2 (5)	C13—C14—C15—C16	−1.3 (7)
N1—C4—C5—C6	−160.3 (3)	C14—C15—C16—C17	1.9 (7)
C10—C5—C6—C7	−1.0 (5)	C15—C16—C17—C12	−1.6 (6)
C4—C5—C6—C7	−176.7 (3)	C15—C16—C17—C19	177.2 (4)
C10—C5—C6—C18	177.4 (4)	C13—C12—C17—C16	0.6 (6)
C4—C5—C6—C18	1.7 (5)	C11—C12—C17—C16	−176.3 (4)
C5—C6—C7—C8	1.9 (6)	C13—C12—C17—C19	−178.2 (4)
C18—C6—C7—C8	−176.5 (4)	C11—C12—C17—C19	4.9 (6)
C6—C7—C8—C9	−2.5 (6)		

### Hydrogen-bond geometry (Å, °)

**Table:**

*D*—H···*A*	*D*—H	H···*A*	*D*···*A*	*D*—H···*A*
C1—H1A···Br1^i^	0.93	2.70	3.531 (4)	149
C2—H2A···Br1^ii^	0.93	2.67	3.579 (4)	165
C3—H3A···N4^iii^	0.93	2.50	3.377 (6)	157
C4—H4B···Br1^i^	0.97	2.86	3.730 (4)	149
C7—H7A···N4^iv^	0.93	2.60	3.390 (5)	144
C10—H10A···Br1^v^	0.93	2.88	3.678 (4)	144

Symmetry codes: (i) −*x*+1, *y*−1/2, −*z*+1/2; (ii) −*x*, *y*−1/2, −*z*+1/2; (iii) −*x*, *y*+1/2, −*z*+1/2; (iv) *x*+1, −*y*−1/2, *z*+1/2; (v) *x*, *y*−1, *z*.
